# Focus on Cognitive Enhancement: A Narrative Overview of Nootropics and “Smart Drug” Use and Misuse

**DOI:** 10.3390/biology14091244

**Published:** 2025-09-11

**Authors:** Fabrizio Schifano, Stefania Bonaccorso, Davide Arillotta, John Martin Corkery, Giuseppe Floresta, Gabriele Duccio Papanti Pelletier, Amira Guirguis

**Affiliations:** 1 Psychopharmacology, Drug Misuse and Novel Psychoactive Substances Research Unit, Hertfordshire Medical School, University of Hertfordshire, Hatfield AL10 9AB, UK; f.schifano@herts.ac.uk (F.S.); j.corkery@herts.ac.uk (J.M.C.); gducciopapantip@gmail.com (G.D.P.P.); 2 North Islington Core Team (N1) North London NHS Foundation Trust, London N7 8US, UK; stefania.bonaccorso2@nhs.net; 3 RCPsych Academic Secretary (London Division), London E1 8BB, UK; 4 Department of Neurosciences, Psychology, Drug Research and Child Health, Section of Pharmacology and Toxicology, University of Florence, 50139 Florence, Italy; 5 Department of Drug and Health Sciences, University of Catania, 95124 Catania, Italy; giuseppe.floresta@unict.it; 6 Tolmezzo Community Mental Health Centre, ASUFC Mental Health and Addiction Department, 33028 Tolmezzo, Italy; 7 Pharmacy, Medical School, The Grove, Swansea University, Swansea SA2 8PP, UK; amira.guirguis@swansea.ac.uk

**Keywords:** cognitive enhancers, smart drugs, drug misuse, psychopharmacology, cognition, nootropics, cognitive dysfunction

## Abstract

Whilst “nootropics” are meant to treat a range of medical disorder-related cognitive impairments, the typically healthy “smart drugs” user ingests a range of drugs/molecules to achieve improved mental performance. Given the increasing levels of related concerns, this study aimed to provide an overview of the clinical pharmacological issues relating to both the most popular nootropics and the vast range of drugs that are being used as putative cognitive enhancers/smart drugs. In terms of the cognitive decline associated with neurological degenerative disorders, a significant variation in research methodology was observed. Therefore, the overall usefulness of these pharmaceuticals in various central nervous system disorders as supplements/adjuvant therapy needs to be better established before their widespread use can be recommended. The most popular smart drugs, self-administered to cope with high-perceived stress and academic/work-related pressure, were methylphenidate, modafinil, amphetamine-based compounds, and psychedelics. At present, however, there are relevant levels of uncertainty in terms of smart drugs’ effectiveness in improving executive functions. Addressing the health harms associated with cognitive enhancers’ intake remains challenging due to the lack of updated and contextualized epidemiological data. In particular, there appears to be a range of clinical concerns relating to the non-prescribed intake of stimulant smart drugs by otherwise healthy individuals. Enhanced training for prescribers, pharmacists, and healthcare professionals can strengthen monitoring and early intervention efforts.

## Simple Summary

While nootropics are designed to treat cognitive problems linked to medical conditions, many healthy people now use “smart drugs” to try to boost their mental performance. This study reviews the clinical issues around popular nootropics and other substances used for this purpose. Research on these drugs, especially for age-related brain disorders, varies a lot, making it hard to say how useful they really are as treatments or supplements. The most commonly used smart drugs—often taken under stress or pressure at school or work—include methylphenidate, modafinil, amphetamines, and psychedelics. However, it is still unclear how effective these drugs are at actually improving thinking and decision-making skills. Health risks from using them, especially without a prescription, are a concern. There is also a lack of recent, detailed data on how many people are using these drugs and what effects they’re having. Better training for doctors, pharmacists, and healthcare workers could help catch problems early and improve safety.

## 1. Introduction

When considering “nootropics”, clinicians aim at treating cognition deficits in patients suffering from Alzheimer’s disease, schizophrenia, stroke, attention deficit hyperactivity disorder, or aging [1,2]. Conversely, the use of “smart drugs” [3] refers to the intake of a range of drugs/molecules to achieve improved mental performance in healthy individuals [4]. Different synonyms include: cognitive enhancers [5]; pharmacological neuroenhancement (PNE) [6]; “study” drugs [7]; and “brain doping” substances [8].

Cognition refers to a capacity for information processing and applying knowledge. It involves memory, attention, executive functions, perception, language, and psychomotor functions (for a thorough overview, see Bayne et al. [9]). In particular, memory has been defined as the ability to remember events or learned material [10]; attention refers to selectively concentrating on one aspect while ignoring distractors; and creativity relates to the ability to create products or ideas that are original [11]. Another important factor is salience [12], describing how prominent or emotionally significant something may be.

### 1.1. Nootropics

Nootropics’ key characteristics may include: increased levels of learning acquisition; increased resistance of learned behaviours against agents that tend to damage them; facilitation of inter-hemispheric flow of information; and increase in the efficacy of tonic cortico-subcortical control mechanisms (for an overview, see Giurgea and Salama [13]. In association with this, nootropics’ indications may include: acute/subacute conditions, e.g., early-stage brain damage and acute psycho-organic syndrome [14]; and chronic cognitive disorders such as Alzheimer’s senile dementia, although they are likely to be most beneficial in mild cognitive disorders as opposed to more severe dementia cases [15]. Other putative indications may include, but are not limited to, attention/memory disorders due to fatigue or exhaustion; minimal brain dysfunction syndrome (children) and encephalopathy; myalgic encephalomyelitis/chronic fatigue syndrome; and schizophrenia-related cognitive decline (for a thorough overview, see Malík and Tlustoš [15]).

Available nootropics are mostly of natural origin (e.g., food supplements, herbal extracts [16]). Conversely, some synthetic forms require a prescription, but most are available without one. Overall, they are well tolerated, with a low incidence of side-effects, which are usually mild [15]. Long-term use may be required for noticeable effects to be clinically perceived [15].

### 1.2. Smart Drugs

Global demand for smart drugs/putative cognitive enhancers (CEs) is booming; they may be available over-the-counter (OTC) in some countries, on prescription, from the internet, from dealers, and through diversion from friends and family (for a thorough overview, see Schifano et al. [3]. Most typically, smart drugs are central nervous system stimulant substances aiming at improving cognitive functions; due to high-perceived stress and academic pressure, CEs are becoming increasingly popular among university students [7]. At present, however, there are relevant levels of uncertainty in terms of smart drugs’ effectiveness in improving executive functions [3]. Furthermore, a range of medical and psychopathological risks may be associated with self-prescribed stimulant medication intake.

In considering the above, this paper aimed at providing an overview of the clinical pharmacological issues relating to both the most popular nootropics and the vast range of drugs that are being used as putative cognitive enhancers/smart drugs. Particular focus is on both the medical and psychopathological disturbances with which they can be associated.

## 2. Methodology

For this narrative review, a literature search was performed, in all languages, using the PubMed (https://pubmed.ncbi.nlm.nih.gov/, accessed on 30 June 2025), Scopus (https://www.sciencedirect.com/topics, accessed on 30 June 2025), and Web of Science (https://clarivate.com/academia-government/scientific-and-academic-research/research-discovery-and-referencing/web-of-science/, accessed on 30 June 2025) databases from inception until March 2025 through the following search strategy: (“nootropics”; or “smart drugs”; or “amphetamine-type substances”; or “Adderall”; or “modafinil”; or “methylphenidate”; or “methylphenidate analogues”; or “Provigil”; or “donepezil “; or “selegiline”; or “cognitive enhancers”; or “piracetam”; or “benzodiazepine inverse agonists”; or “unifiram”; or “Ginkgo biloba”; or “piracetam”; or “caffeine”; or “Guarana”; or “Maca”; or “Ashwagandha”; or “Ginseng”; or “phosphatidylcholine”; or “naftidrofuryl”; or “dihydro-ergotoxine”; or “vinpocetine”; or “pyritinol”; or “nicergoline”; or “meclofenoxate”; or “deanol”; “aniracetam”; or “oxiracetam” and “healthy individuals”; and “Alzheimer dementia” and “cognitive decline”; and “cognition”; and “acute psycho-organic syndrome”; and “use”; and “misuse” and “intoxication”). Particular focus was also on the medical and psychiatric manifestations associated with the above substances. Evidence included in the review comprised both human data and preclinical data, if and when this was of relevance. Each article’s title and abstract were reviewed by A.G. and F.S. for their appropriateness with regard to the relationship between cognitive enhancement and the specific classes of substances highlighted here. Although papers quoted here were published in the time frame 1977–2025, about half of them appeared in the 2020–2025 period.

## 3. Results

### 3.1. Nootropics

Overall, nootropics are thought to improve brain function without directly releasing neurotransmitters or binding to receptors. Indeed, they should facilitate enhancement of both glucose and oxygen supply to the brain; provide anti-hypoxic effects and protect against neurotoxicity; stimulate neuronal protein and nucleic acid synthesis; promote phospholipid metabolism in neurohormonal membranes. Furthermore, they could facilitate elimination of oxygen-free radicals; improve blood flow; and enhance erythrocyte plasticity (for a thorough review, see Malík and Tlustoš [15]). All nootropics will arguably need to penetrate the blood–brain barrier (BBB) for optimal brain metabolism; whilst being suggested to be metabolically active, they require long-term use to show effects [17].

A range of both natural and synthetic nootropics have been identified. Typically, natural nootropics are sourced from plant parts (flower, leaf, root) and offer diverse and synergistic effects due to varied composition [18,19]. One would argue, however, that some natural compounds require high doses for effectiveness; and there are issues with storage, authenticity, and falsification. Conversely, synthetic nootropics may well provide pharmaceutical purity and specificity of action; can be chemically modified to enhance effects; and are effective at lower doses, although higher toxicity risks may be observed [15].

#### 3.1.1. Mechanism of Action

Regarding the nootropics’ mechanism of action, several explanations have been proposed, including:

#### 3.1.2. Shift in Energy Metabolism

Due to limited success with neurotransmitter-based approaches, research has focused on modifying neuronal metabolism. From this point of view, piracetam [20] intake has been associated with increasing levels of adenylate kinase activity; enhancement of 32P uptake in neuronal and glial cells; improved glucose utilization under low O_2_ conditions; and speeding up of EEG recovery. Conversely, no increase in glucose level utilization was observed with piracetam [20,21].

#### 3.1.3. Cholinergic Effects

Following the better understanding of the role of acetylcholine (ACh) in Alzheimer’s disease (SDAT) [22], nootropic-based research focus eventually shifted to the role of this neurotransmitter. Indeed, piracetam was shown to increase both choline uptake and cholinergic receptor density in the frontal cortex, and oxiracetam prevented decreases in ACh levels in the cortex and hippocampus following electro-convulsive treatment procedures in animals. Findings across studies were overall inconsistent, and the evidence for cholinergic mechanisms in nootropics remains unreliable and contradictory [21].

#### 3.1.4. Involvement of Excitatory Amino Acids

New research focuses on long-term potentiation (LTP) in glutamate transmission. LTP may have a role in enhancing receptor-mediated activity through α-amino-3-hydroxy-5-methyl-4-isoxazolepropionic acid (AMPA) receptor regulation. From this point of view, it has been suggested that oxiracetam may partially counteract behavioral effects of NMDA receptor antagonists whilst increasing glutamate release in hippocampal slices [23]. It is unclear, however, if the effects of aniracetam are the same as those produced by LTP.

#### 3.1.5. Steroid Involvement

A steroid involvement in nootropic effects has been postulated as well, since there may be possible internal memory-enhancing mechanisms that trigger “flashbulb memories” [24]. Furthermore, a range of animal studies suggested that adrenalectomy nullified memory-boosting effects of steroids and ACTH, though learning capacity remained unaffected [25]. Furthermore, aminoglutethimide, which may counteract the adrenal cortex functioning, rendered piracetam-like nootropics ineffective [26].

Additional mechanisms have been suggested to explain the nootropics’ putative therapeutic effectiveness, including their associated elevated cAMP levels and ATP/ADP ratio [27]; amplified brain metabolism via oxidative catabolism [28]; and enhanced phospholipid metabolism, protein biosynthesis, and ion flux modulation [29].

Generally, nootropics are well tolerated, being associated with only typically mild side-effects, consisting of sleep disturbances; increased libido; and undesired heightened activity [15]. Their clinical effectiveness may depend on dosing, and treatment should continue for 2–3 weeks after those consciousness disturbances for which they were initially considered resolve. Contraindications include hypersensitivity; pregnancy; and lactation [15].

#### 3.1.6. Nootropics’ Classification

Nootropics can be tentatively grouped as synthetic molecules; vasodilatory substances increasing brain metabolism; and compounds of natural origin (for thorough reviews see Napoletano et al. [30]; Froestl et al. [1,2]).

(a) Classical synthetic nootropics

These compounds include (for an overview, see Malík and Tlustoš [15]; Mondadori [31]) a range of synthetic compounds that have been explored for their cognitive-enhancing properties, many of which act on cholinergic pathways or cerebral metabolism. For instance, deanol (DMAE) and meclofenoxate serve as choline precursors, supporting acetylcholine synthesis and potentially improving learning and memory, with suggested daily intakes ranging from 500 to 2000 mg. Nicergoline, an ergot derivative, is thought to improve cognitive function, mood, and alertness and is typically administered at 30–60 mg/day. Piracetam, a well-known nootropic, modulates calcium and potassium ion channel activity, enhancing neuronal excitability; it is used acutely at 4–8 g/day, with maintenance doses between 2 and 4 g/day. Pyritinol, a derivative of vitamin B6, may enhance choline acetyltransferase activity and is administered at a minimum of 300 mg/day, though higher doses (>600 mg/day) are often used clinically.

(b) Cerebral Metabolism Enhancers

Some compounds possess overlapping nootropic, vasodilatory, and hemorheological properties. Vinpocetine, for example, is thought to enhance cerebral oxygenation and is initiated at 2–5 mg/day, increasing to 10–30 mg. Naftidrofuryl improves peripheral circulation and blood rheology with doses of 300–600 mg/day.

(c) Natural; Plants and Their Extracts with Nootropic Effects

The group contains a range of compounds (for thorough reviews see Malík and Tlustoš [15,32] and Graziano et al. [33]). Several botanical extracts are associated with cognitive enhancement. Phosphatidylcholine, a key component of lecithin, a substance found in various plant and animal sources lecithin serves as a choline reservoir. Therapeutic efficacy in older adults may require significantly higher doses, with preventative use around 3.6 g/day and therapeutic doses exceeding 10–15 g/day. Dihydroergotoxine, a composite of ergot alkaloids, has shown promise in managing cognitive decline and protecting neural tissue from hypoxic damage, with a recommended dose of up to 6 mg/day.

Ginseng (*Panax ginseng*) has shown benefits in memory, in patients with typical dosages used being 200 mg/day of standardized extract (containing 1.5–7% ginsenosides) or 0.5–2 g/day of dried root. Most studies have focused on the actions of *P. ginseng* ginsenosides, which may be responsible for the effects on the nervous system. Further potentially bioactive ingredients include phytosterols, sesquiterpenes, flavonoids, polyacetylenes, alkaloids, and phenolic compounds [34]. Ginseng can influence the monoamine neurotransmitter system; increase the expression of neurotrophic factors like BDNF; and interact with various ion channels (Ca2+, K+, Na+) and ligand-gated ion channels (GABAA, 5-HT3, nicotinic acetylcholine, and NMDA receptors), potentially stabilizing excitable cells and modulating neuronal activity [35]. *Ginkgo biloba* exhibits antioxidant and vasodilatory properties and is potentially beneficial for patients with cerebral insufficiency or neurodegenerative disorders. It is typically used with standardized extract doses (EGb 761^®^) ranging from 120 to 300 mg/day. Mechanisms of action include increasing cerebral blood flow, antioxidant and anti-inflammatory effects, with antiplatelet effects attributed to flavone and terpene lactones [36]. Improved levels of circulation can enhance oxygen and nutrient delivery to brain cells, potentially mitigating the effects of ischemia and promoting cognitive function, whilst flavonoids and terpenoids can protect brain cells from oxidative damage. In preclinical studies, flavonol derivative ingestion has been associated with increases in both dopaminergic and cholinergic neurotransmission in the prefrontal cortex [37].

Ashwagandha (*Withania somnifera*) may offer neuroprotective benefits, including in neurodegenerative conditions, e.g., Alzheimer’s disease or tardive dyskinesia, at dosages of 750–1250 mg/day of extract or 6–10 g/day of ground root. The leaf extract and its component withanone protect from scopolamine-induced toxic changes in both neuronal and glial cells. *W. somnifera* extract attenuated lead-induced toxicity in glial cells; glycowithanolides exhibited significant antioxidant activity in cortex and striatum of rat brain; and its root extracts/derivatives promoted neurite outgrowth extensions in human neuroblastoma cell lines (for a thorough overview, see Sprengel et al. [38]). Furthermore, Ashwagandha may be associated with increased expression of sleep-related GABA_A_, GABA_B_, and 5HT_1A_ and an overall increase in brain GABA content [38].

Guarana (*Paullinia cupana*), rich in caffeine (~12% in extract), hence primarily exerting its effects through its stimulant properties, is linked to enhanced memory and physical performance, though it is contraindicated in cardiovascular conditions. Finally, Maca, often referred to as “Peruvian ginseng”, in being an adaptogen [39], has been studied for its potential to boost cognitive and motor performance, with doses typically in the range of 1.5–3 g/day.

### 3.2. Smart Drugs

#### 3.2.1. Mechanism of Action

Most typically, smart drugs are represented by a range of psychostimulants, whose main action involves action on the dopaminergic (DAergic) neurotransmitter pathways. Indeed, DA is involved in a number of cognition-related areas, including salience; attention; perseverance/determination; motivation; working memory; decision making; and the ability to resist higher than the usual levels of intellectual challenges [40]. An optimal cognitive function may require, however, a balanced level of dopamine; too little can lead to difficulties with focus and motivation; conversely, excessive central DA levels in the mesolimbic/mesocortical pathways can result in impulsive behavior; paranoid ideation; and impaired cognitive control (for thorough overviews of the role of dopamine in cognition, see Westbrook and Braver [41] and Cools et al. [42]).

#### 3.2.2. “Smart” Drugs Popularity as “Study” Drugs

Due to the above pharmacodynamic characteristics, smart drugs/CEs are particularly appealing to students, hence at times being referred to as “study” drugs. According to Sharif et al. [43], who carried out a survey in a range of schools from several United Arab Emirates (UAE) universities, CE users were disproportionately (*p* < 0.001) represented by students of medicine, followed by pharmacy, dentistry, and engineering with respect to the other schools. In confirming this, some 1399 medical students from Egypt, Sudan, and Jordan were surveyed, and some 1236 (88.3%) admitted to being CE consumers [7]. Nowrouzi and Richelle (2024) interviewed 323 final-year medical students [44]. It seemed that these students’ CE use increased over the years, with some 12.6% of final-year pupils reporting that they had already used CEs, and 3.6% were currently using them. Associated risk factors included sensation seeking, other substances’ use, high stress levels, social influence, and lack of ethical concern regarding substance use. A CEs’ use online questionnaire was completed by 1156 Portuguese medical students/newly qualified physicians; methylphenidate (MPH; 35%) and modafinil (10%) were the most popular study drugs being ingested [45]. Similarly, some 1453 Israeli medical residents were surveyed; 28.1% of responders reported past CEs’ use, with 73.67% of them reporting use without a related medical diagnosis [46]. Finally, 370 Israeli resident physicians responded to a CEs’ use questionnaire; 16.4% were classified as users and 35.1% as misusers. Very high levels of MPH misuse during residency were allegedly related to stress and long working hours [47]. Overall, according to Sharif et al. [48], students from highly competitive programmes, requiring top grades from applicants for entry, would be at particular risk of misusing CEs. This may be due to both the unusual levels of hard academic work required and the high tuition fees involved, and hence the perceived need to academically perform at the highest level [48].

#### 3.2.3. Smart Drugs in Sports and Warfare

Smith et al. (2020) discussed how the use of this class of molecules in sports could be associated with improved focus, concentration, alertness, and rapid decision making; however, the health implications of cognitive-enhancing drugs in this area remain unclear [49]. Taking substances to enhance the brain is more popular among amateur athletes than taking drugs to boost the body; some 15% of 2997 recreational triathletes admitted to brain doping [50]. Furthermore, in baseball, up to 8% of major league players have been apparently diagnosed with attention deficit hyperactivity disorder (ADHD) and prescribed related stimulant medications [51]. Smart drugs could be particularly appealing as well to professional video gamers and other “mind sports/e-athletes” [52].

Conversely, there have been suggestions of the use of some compounds in warfare. For example, “Captagon” (e.g., phenethylline, a synthetic stimulant combining amphetamine and theophylline) has been associated with better focus; determination; increased levels of resilience/perseverance; lack of empathy; and ideas of reference which are very useful to escape from possible threats [53,54]. Furthermore, according to Malish et al. [55], some military forces are embarking on a strategy designed to produce significant shifts in the future of warfare; this strategy, however, will need to include American operators’ competitive edge in cognition. This may entail the use of therapeutic agents to enhance human–machine interplay, with the aerospace medical community representing an ideal candidate to carry out this work [55].

#### 3.2.4. Most Popular Smart Drugs

Most popular smart drugs include methylphenidate; modafinil; amphetamine-based compounds; psychedelics; and a miscellany of other molecules/novel psychoactive substances (NPS).

(a) Methylphenidate; Modafinil; Amphetamine-Based Compounds; Psychedelics

Methylphenidate

In May 2016, the “Oxford student” newspaper published a survey showing that 15.6% of students knowingly took a range of stimulants, including methylphenidate, without a prescription [56]. A large number of methylphenidate-related compounds have been described, including ethylphenidate, 3,4-dichloromethylphenidate, 3,4-dichloroethylphenidate, 4-fluoromethylphenidate, 4-fluoroethylphenidate, methylnaphthidate, ethylnaphthidate, isopropylphenidate, propylphenidate, 4-methylmethylphenidate, and N-benzylethylphenidate [57,58,59].

Modafinil

This Food and Drug Administration- and European Medicines Agency-approved molecule directly increases cortical catecholamine levels, indirectly upregulating cerebral serotonin, glutamate, orexin, and histamine levels, and indirectly decreasing GABA levels [60]. Modafinil was developed to treat narcolepsy, but it is widely used off-license as a smart drug to promote cognitive enhancement due to alleged increased levels of alertness and concentration [61]. During exam time, the volume of web-based modafinil being shipped to Cambridge; Oxford; Imperial College; and the London School of Economics may double. Students tend to take modafinil rather than either Ritalin or Adderall [62]. Overall, most studies employing basic testing paradigms suggested that modafinil intake enhances executive functions; only half of literature reports showed improvements in attention and learning and memory, whilst a few reported impairments in divergent creative thinking (for an overview, see Schifano et al. [3]). In contrast, when structured research protocols are used, modafinil may appear to consistently facilitate enhancement of attention, executive functions, and learning [63].

Amphetamine-Type Substances’ (ATS) Mixtures; Adderall

Adderall, used to treat attention deficit hyperactivity disorder (ADHD) and narcolepsy, contains a combination of amphetamine and dextroamphetamine. Adderall/amphetamine salt mixture was identified as the most commonly misused CE in 6 highly competitive UAE courses [48]. Apart from Adderall, amphetamine-type substances include oral (“meth”), smokable (“crystal meth”), and injectable/rectally administered methamphetamine, together with a vast range of other substances, including PMA (4-methoxyamphetamine, “Dr. Death”), PMMA (4-methoxymethamphetamine), 4-MTA (4-methylthioamphetamine, “flatliners”), DMA (2,5-dimethoxyamphetamine), MPA (methiopropamine), etc. [57]. Indeed, there are differences in bioavailability levels between the different methamphetamine formulations; when injecting the molecule, the bioavailability (BA) levels will equal 100%; with the smokable formulation, the BA levels exceed 90%, and with oral meth, the related BA levels may be around 60% [64]. Most typically, however, ATS are ingested orally.

Rationale for Stimulant use

Stimulants (including methylphenidate; modafinil; and ATS) act on DA neuron terminals projecting from the ventral tegmental area to the nucleus accumbens, representing the reward system in the brain [65]. Stimulant drugs are strong DA agonists, hence, arguably, increasing attention; memory; motivation; focus; salience; and coping with challenging academic tasks [66].

Risks Associated with Stimulant use

Stimulant ingestion may be associated with euphoria, an urge to re-dose, and a sense of full energy but also a significant rise in body temperature and death; fatalities are particularly associated with seizures and coma [67]. The main action of stimulants is on the cardiovascular system, due to their short- and long-term stimulation of the adrenergic system and consequent effects on blood pressure and myocardial ischemia (for a thorough review, see Duflou [68]). Conversely, chronic ingestion of stimulant-related drugs is typically associated with dependence, chronic depression, anxiety, tendency to suicide, drug-related psychosis [69], myocardial infarction, hypertension, stroke, psychosis, mood disorders, and death [57,67].

(b) Psychedelics’ Microdosing

When being considered as smart drugs, psychedelics are typically ingested in “microdoses”. Microdosing involves the intake of sub-threshold doses of LSD, psilocybin, or dimethyltryptamine (DMT) on 2–4 occasions per week; this may be carried out for weeks/months/years [70].

MDMA has been microdosed as well [71]. Psilocybin is likely to be the most common drug being microdosed (70%), followed by LSD (57%). Among microdosers, ~18% reported, however, using psychoactive doses far higher than would be generally considered a microdose [72].

During the 1960s, LSD was typically ingested at a 120–150 μg dosage [73]. When microdosed, perceptible drug effects may be reported at doses of 10 to 20 μg but not 5 μg [74]. It is worth noting that, at low dosages, LSD may act more as a stimulant than as a psychedelic. Consistent with this, with respect to placebo LSD, taken in microdoses, it may activate the DAergic reward pathways and increase motivational levels [75].

Syed et al. [76] surveyed some 6193 psychedelic consumers; 2488 were microdosers, reporting having had previous experience with up to 11 different classical and atypical psychedelics. Most consumers microdosed psychedelics multiple times a month; popular molecules included psilocybin (74.5%), LSD (34.4%), and ketamine (15.8%) [76]. A convenience sample of 736 UK students/staff were surveyed in relation to their CE use during the first year of COVID social restrictions compared with the previous year. A significant self-reported rise in the use of all drug types (e.g., all at  <0.001) during social restrictions was observed; rising levels were particularly observed with modafinil (+42%), nutraceuticals (+30.2%), and microdose LSD (+22.2%; [77]). Murphy et al. [78] assessed microdosing’s effects on creativity; 80 healthy adult males were given 10 µg doses of either LSD or placebo every third day for six weeks, e.g., 14 total doses. Although feeling more creative on dose days, objective measurement found no acute or durable effects of the microdosing protocol on creativity [78]. Overall, placebo-controlled studies have failed to demonstrate strong cognitive microdosing benefits. Stimulatory effects of LSD may be strongest in those with low arousal and attention at baseline, while inhibitory effects were strongest in high memory performers at baseline [79]. Notably, a variety of edible products containing psilocybin or psilocyn (e.g., chocolate products containing mushrooms) are emerging on the market; their inappropriate use has been recently reported, and in some cases serious effects have been observed after ingestion [80].

(c) Other Smart Drugs

A diverse range of molecules have been anecdotally mentioned for their cognitive enhancement purposes.

Selegiline

Some report that the long-term use of a 5 mg daily dose of selegiline helps in boosting mood, energy, and libido [3]. Indeed, (-)deprenyl (e.g., selegiline, an antiparkinsonian agent at times used in some countries as an antidepressant) is metabolized to L-amphetamine and L-methamphetamine [81]. Selegiline is a selective monoamine oxidase (MAO)-B inhibitor at lower doses and also of MAO-A at higher doses, while preventing the reuptake of norepinephrine, serotonin, and dopamine in the CNS. Furthermore, the positive clinical effects of selegiline may be associated with both reduction in neuronal apoptosis [82] and with activation of growth factors [83]. Although selegiline-related dopaminergic/noradrenergic enhancement may facilitate cognitive recovery in preclinical studies [84], no randomized controlled trials have confirmed any putative neuroenhancement effects of selegiline in healthy adults.

Benzodiazepine Inverse Agonists

Although not associated with the related anxiety/agitation common with other non-selective inverse agonists, some GABA-A receptor α5 selective inverse benzodiazepine agonists have been reported as possessing neuroenhancement properties [85]. In carrying out a range of docking/in silico studies, Catalani et al. [86] identified some 12 “designer benzodiazepine” molecules putatively possessing GABA-A receptor α5 selective inverse agonist activities; these included fluadinazolam, pyclazolam, pynazolam, and tofisopam. Their overall mechanism remains unclear, although one could argue about their possible DAergic agonist potential [86].

Unifiram and Its Analogues

Unifiram, sunifiram, and sapunifiram are reported, in web-based discussions, as smart drugs. They were identified, some 45 years ago, as putative, not patent protected, CEs [87]. They are structurally related to ampakines [88,89] and may possess anti-amnesic effects [3]. However, no data regarding a possible global scale of their usage and/or their prevalence or patterns of use are available at present.

## 4. Discussion

An updated, narrative overview of the pharmacological, clinical pharmacological, and toxicity issues related to the variety of molecules being either prescribed or self-administered/misused to obtain significant levels of cognitive enhancement is provided here. In considering that half of the current papers quoted here were published in the 2020–5 timeframe, one could argue that there is recent, further increased interest in these molecules.

Overall, the range of putative nootropics/cognitive enhancers/smart drugs/study drugs appeared to be large and diversified. Napoletano et al. [30] aimed at identifying and better describing, with a mixed-methods, web-based, netnographic study, the range of these molecules. They mentioned a total of 142 unique CEs, which were ranked into 10 categories, including plants/herbs/products (29%), prescribed drugs (17%), image and performance enhancing drugs (IPEDs) (15%), psychostimulants (15%), miscellaneous (8%), phenethylamines (6%), GABAergic drugs (5%), cannabimimetics (4%), tryptamine derivatives (0.5%), and piperazine derivatives (0.5%) [30].

Nootropics appeared here to represent a heterogeneous group of drugs affecting neuronal metabolism in the CNS. They may improve cognitive function, especially in cases of damage or degeneration, but they could require prolonged use for measurable effects. Their clinical indications may be related to memory, consciousness, and learning disorders. Furthermore, nootropic agents, which are generally well tolerated, with side effects being rare and mild, may have a supporting role for degenerative diseases [90].

Conversely, most smart drugs are self-administered by otherwise healthy, bright individuals with typically either an academic or a high-rank job involvement [48]. Interestingly, it has been suggested that “stimulant” smart drug users are likely to possess antisocial characteristics and are indifferent to rules, whilst “depressant” CE drug users may be more motivated by coping with stress [91]. With most smart drugs being potent DAergic stimulants, one could, however, argue that the related modulation of central noradrenaline, glutamate, and dopamine levels may lead to cardiovascular, neurological [5], and psychopathological complications [57]. Furthermore, smart drugs’ use can be associated with paradoxical short- and long-term cognitive decline and addictive behavior [3].

The use of sustained high doses of cognitive enhancers by healthy individuals may yield some desired effects; however, this is often accompanied by an increased risk of adverse outcomes. In individuals without cognitive impairment/deficit, the potential for cognitive enhancement is limited, as their baseline function is already near optimal. Consequently, rather than achieving a “supernormal” boost, these individuals are more likely to experience negative effects, including sleep disturbances, mood instability, anxiety, reward-system dysfunctions/addiction behaviors, and other psychopathological symptoms. The risk-benefit ratio in healthy populations might be, therefore, unfavorable, particularly with long-term or non-medically supervised use.

From Clinical to Cosmetic Psychopharmacology?

According to Lugg [92], cosmetic psychiatry may be defined as “the science and practice of interventions that subjectively enhance the mental states of healthy people”. Whilst cosmetic medicine/surgery is indeed a professionally appropriate component of clinical practice, cosmetic psychiatry is not [92]. Indeed, there are significant risks associated with cosmetic psychiatry, and this may be especially true for cosmetic psychopharmacology [93]. One could indeed argue about the possible medical and psychopathological consequences being possibly triggered in those students/professionals who present with normal/higher than the norm cognitive levels but who are ingesting, on a long-term basis, a psychedelic and/or a potent stimulant. Excessive and sustained 5-HT2A and DA activation are associated with medical and psychopathological disturbances, even in those subjects without a previous psychiatric history [57]. Apart from the possible health-related consequences, are there ethical concerns relating to the long-term retention of any “on drug” acquired knowledge? Indeed, there is an urgent need for a broader discussion about this emerging clinical reality [92].

## 5. Conclusions

Addressing the health harms associated with smart drug/nootropic/cognitive enhancer intake remains challenging due to the lack of updated and contextualized epidemiological data. Overall, there appear to be a range of clinical concerns relating to the non-prescribed intake of these molecules by otherwise healthy individuals, given the insufficient levels of clinical evidence relating to their effectiveness as well in healthy users. From this point of view, a diverse range of participant groups (e.g., in terms of age, gender, health status, and occupation/discipline), with a particular focus on young, healthy users, will need to be considered in the future. There should also be increased use of neuroimaging for confirming any possible cognitive effects associated with the effects of these drugs. Even in terms of the cognitive decline associated with degenerative disorders such as Alzheimer’s dementia, a significant level of variation in research methodology was observed. Therefore, the overall usefulness of these pharmaceuticals in various central nervous system disorders as a supplement or adjuvant therapy needs to be established through high-quality multicenter randomized controlled clinical trials with sufficient sample size and optimized study design before their widespread use can be recommended [17].

Moreover, a growing concern is the emergence of designer drugs, which are synthetically modified substances created to mimic the effects of controlled drugs whilst avoiding legal restrictions. These compounds are often engineered with subtle molecular modifications that may significantly alter pharmacological properties and toxicity profiles, posing serious risks to users [94,95,96]. The rise of AI-driven molecular design tools could further accelerate the development of NPS, making detection and regulation increasingly complex. Finally, future health policy interventions will need to be informed by the establishment of a corpus of data relating to the patterns and consequences of smart drug use. Enhanced training for prescribers; pharmacists; and healthcare professionals can strengthen monitoring and early intervention efforts. A multidisciplinary approach that combines medical, psychological, and social support remains key to managing the complex challenges posed by smart drug use.

## 6. Limitations

In order to fulfill the purpose of this review, presenting with a focus on a very specific topic with most of the studies being relatively novel, a narrative approach was preferred. This choice, which made explicit the search strategy, has anyway provided broader literature coverage and more flexibility. We acknowledge, however, that this design has introduced several limitations. First, some of the studies included here did not disclose explicit criteria for article selection, which could lead to potential selection bias. Second, the review strategy employed led to the inclusion of studies with highly heterogeneous designs, methodological quality, and standardization of the methods used to characterize the impact of specific substances on cognitive function. Some of the included studies were small-scale, and, therefore, it remains unclear whether the findings could be generalized to the wider population of smart drug enthusiasts. Finally, the literature collected was variously referring to “smart drugs”; “nootropics”; or “cognitive enhancers”, hence creating levels of difficulties in data interpretation.

## Data Availability

No new data were created or analyzed in this study. Data sharing is not applicable to this article.
